# Towards a standardization of thrombin generation assessment: The influence of tissue factor, platelets and phospholipids concentration on the normal values of Thrombogram-Thrombinoscope assay

**DOI:** 10.1186/1477-9560-3-16

**Published:** 2005-10-26

**Authors:** Grigoris T Gerotziafas, François Depasse, Joël Busson, Lena Leflem, Ismail Elalamy, Meyer M Samama

**Affiliations:** 1 Service d'Hématologie Biologique, Hôpital Hôtel-Dieu de Paris, France; 2 LCL, Ivry sur Seine, France

**Keywords:** Thrombin generation, Thrombinoscope, endogenous thrombin potential, thrombin, platelets, tissue factor

## Abstract

**Background:**

Thrombin generation assay was developed several years ago to mimic physiological coagulation mechanisms but it had important limitations. Thrombogram-Thrombinoscope assay using a fluorogenic substrate, allows obtaining thrombin generation curves in non-defibrinated platelet rich plasma (PRP) in a fully automated manner.

**Methods:**

We standardised the methodology of Thrombogram-Thrombinoscope and we evaluated the precision of thrombin generation parameters (lag-time, maximum concentration of thrombin [Cmax], time required to reach Cmax [Tmax] and endogenous thrombin potential ETP) using different concentrations of recombinant human tissue factor, platelets or phospholipids. Normal values of thrombin generation assay were established in optimal experimental conditions.

**Results:**

In the presence of low TF concentrations (final dilution of thromboplastin in plasma: 1/1000–1/2000) the Thrombogram assay showed intra-assay and inter-assay coefficients of variation lower than 9%. Thrombin generation parameters showed an important inter-individual variability and the coefficients of variation ranged from 18% to 50%. In PRP the lag-time, Cmax and Tmax but not the ETP, were influenced by TF concentration. Thrombin generation parameters were not influenced by variations of platelet concentration from 50 × 10^9^/l to 400 × 10^9^/l. The addition of synthetic procoagulant phospholipids in PPP strongly influenced all the parameters of thrombogram. For all the parameters of thrombogram a threshold effect was observed in the presence of phspholipid concentrations equal or higher to 4 μM. In frozen-thawed PRP the lag-time and the Tmax were significantly reduced and the Cmax was increased compared to the fresh PRP, but the ETP, the intra assay and the inter-assay coefficients of variation were similar in both test-systems.

**Conclusion:**

Thrombogram-Thrombinoscope assay performed in fresh or in frozen-thawed PRP has an acceptable precision, with low inter-assay and intra-assay coefficient of variations. The concentration of TF is determinant for the normal values of the studied parameters of thrombin generation. When the assay is performed in PPP, thrombin generation parameters are influenced by the concentration of procoagulant synthetic phospholipids. The optimal experimental conditions were obtained in the presence of 1/1000 final dilution of thromboplastin, a platelet count higher than 50 × 10^9^/l and a synthetic phospholipid concentration higher than 4 μM.

## Background

The global clotting times (i.e. prothrombin time and partial thromboplastin time) are routinely used for the evaluation of human coagulation but they ignore the procedure of thrombin generation since at the time of clot formation only 3% of prothrombin is activated [1,2]. This is a constant observation either in normal plasma or in the presence of antithrombotic agents (i.e. enoxaparin or fondaparinux) [3]. The global clotting times are mandatory for the detection of clotting factor deficiencies and the biological monitoring of treatment with coumarins and unfractionated heparin but they have a very limited value in the detection of hypercoagulable states [1,2]. Moreover, they are not significantly influenced by the presence of therapeutic concentrations of LMWHs or the indirect FXa inhibitors such as fondaparinux [3]. Measuring of anti-Xa activity in plasma from patients treated with LMWHs has a limited predictive value for the clinical outcome [4] and in some patients' populations (i.e elderly or obese patients) the anti-Xa levels in plasma are poorly correlated with the administered dose of LMWH [5,6].

The study of thrombin generation performed either with clotting based assays or with chromogenic substrates is an old and established tool in blood coagulation research [7-12]. It describes all the phases of thrombin generation process (initiation, amplification and inhibition of thrombin generation as well as the integral amount of generated thrombin). According to the experimental system used, thrombin generation may be influenced by most of the factors playing a role in blood coagulation. However, thrombin generation assessment used to be a laborious and time-consuming method. The most sophisticated version of thrombin generation assay using a chromogenic substrate developed by Hemker's group was less time-demanding than the previous one, since it was fully automated accompanied with a software for the calculation of the area under thrombin generation curve (endogenous thrombin potential) [13]. The most important experimental limitation of this assay was that it could be done only in defibrinated platelet poor plasma (PPP). Thus at least two important components of blood coagulation (i.e. platelets and fibrinogen/fibrin) were absent [14].

The Thrombogram-Thrombinoscope assay is a step forward to the study of the blood coagulation process because, using a fluorogenic substrate, it allows obtaining thrombin generation curves in non-defibrinated platelet rich plasma (PRP) in a fully automated manner [15]. An increasing body of evidence shows that Thrombogram-Thrombinoscope assay may be a useful tool in the diagnosis of acquired or congenital hypercoagulable states and it is sensitive enough, in patients with hemorrhagic diathesis [16, reviewed in 17]. Thrombogram is also sensitive to the presence of antiplatelet and antithrombotic agents [3,18].

Until now, there is an important heterogeneity regarding the pre-analytical conditions and the experimental systems for the assessment of thrombogram. In addition, the endogenous thrombin potential is the sole parameter of thrombogram that has been systematically validated [19-21]. Other parameters of thrombin generation curve given by Thrombogram-Thrombinoscope^®^, such as the lag-time of thrombin generation onset, the maximum concentration of generated thrombin and the time required to reach the maximum concentration of thrombin have not been systematically validated.

Standardization of thrombin generation using the Thrombogram-Thrombinoscope^® ^method is necessary towards the wide clinical use of the assay. The aim of this study was to determine the influence of preanalytical and experimental conditions on the Thrombogram-Thrombinoscope assay. We examined the intra-assay, the inter-assay and the inter-individual coefficients of variations of thrombin generation lag-time, the maximum concentration of thrombin (Cmax), the time required to achieve maximum thrombin generation (Tmax) and the endogenous thrombin potential (ETP) in fresh PRP and in frozen-thawed PRP after triggering tissue factor (TF) pathway in plasma from healthy volunteers and we established the normal values for each parameter of the thrombogram. We also studied the influence of experimental condition such as the TF, concentration, and the platelet counts or phospholipids concentration on each parameter of thrombin generation curve given by the thrombogram.

## Methods

### Reagents

Bovine serum albumin (BSA) and Tris-HCl were obtained from Sigma laboratories (St. Louis U.S.A.). Relipidated recombinant tissue factor (Hemoliance^® ^RecombiPlasTin) was from Instrumentation Laboratory (Milan, Italy). The Hemoliance^® ^RecombiPlasTin reagent was reconstituted by addition of 5 ml of NaCl 0.9%, and subsequently diluted in Hepes buffer; pH 7.35 (containing 20 mM Hepes, 140 mM NaCl and 5 mg/ml BSA) for the experiments with the Thrombogram-Thrombinoscope™ assay (Synapse b.v., Maastricht, The Netherlands). Thrombin calibrator was also obtained by Synapse b.v. (Maastricht, The Netherlands). The fluorogenic substrate Z-Gly-Gly-Arg-AMC was obtained from Bachem (Bubendorf, Switzerland). Unfractionated heparin was from Leo Laboratories, commercially available enoxaparin preparation was from Sanofi-Aventis Laboratories (Paris, France) and fondaparinux was from Glaxo Smith Klein (Paris, France). Synthetic phospholipids (DOPS/DOPE/DOPC reconstituted according to manufacturer's instructions in a ratio 1:1:1,25) were obtained from Avanti Polar LipidsInC (Albaster, Alabama, USA).

### Normal human plasma

Venous blood was obtained from 22 healthy volunteers non taking any medication, who are working in our laboratory. Blood was collected with atraumatic antecubital veinipuncture, into siliconized vacutainer tubes (Becton Dickinson, Meylan, France) containing buffered trisodium citrate (0.129 mol/l, nine parts of blood and one part of citrate solution). Thrombogram-Thrombinoscope™ assay was performed in platelet rich plasma (PRP) prepared after centrifugation of citrated whole blood for 10 min at 150 g at room temperature. The supernatant PRP was removed and the platelet count was adjusted to 150 × 10^9^/l by dilution with autologous PPP obtained after a further centrifugation of the remaining blood for 15 min at 2000 g. To study the influence of platelet concentration on thrombin generation parameters, the platelet concentrations in PRP, prepared from 4 healthy volunteers, were adjusted to, 10 × 10^9^/l, 50 × 10^9^/l, 100 × 10^9^/l, 150 × 10^9^/l, 400 × 10^9^/l by dilution with autologous PPP. To study the influence of phospholipids concentration on thrombin generation assay, frozen-thawed PPP from three normal volunteers was spiked with increasing concentrations of synthetic procoagulant phospholipids (0, 2, 4 and 8 μM).

### Thrombogram-Thrombinoscope assay

In each well of a micro-plate, 80 μl of the studied plasma were mixed with 20 μl of diluted relipidated recombinant tissue factor (Hemoliance^® ^RecombiPlasTin) from Instrumentation Laboratory (Milan, Italy). The RecombiPlasTin reagent was reconstituted by addition of 5 ml of NaCl 0.9%, and subsequently diluted in Hepes buffer pH 7.35; containing 20 mM Hepes, 140 mM NaCl and 5 mg/ml bovine serum albumin. The concentration of TF is not given by the manufacturer and for this reason TF is expressed as final dilution of recombiplastin in the plasma. The following final dilutions of recombiplastn were studied: 1/200, 1/500, 1/1000, 1/2000 which correspond to a range of calculated TF concentration from 3 pM to 30 pM in plasma (TF levels in RecombiPlasTin reagent were measured using an ELISA assay from American Diagnostics Inc; data not shown). No inter-lot variability of recombiplastin preparations was observed (data not shown). Thrombogram was also performed without any addition of TF.

Thrombin generation was triggered by adding a solution containing CaCl_2 _(16.7 mM final concentration) and the fluorogenic substrate (Z-Gly-Gly-Arg-AMC, 417 μM final concentration). A plate reader fluorometer (Fluoroskan Ascent, ThermoLabsystems, Helsinki, Finland) and the appropriate software (Thrombogram-Thrombinoscope™ assay; Synapse b.v., Maastricht, The Netherlands) were used. Generated thrombin was quantified using a thrombin calibrator kindly offered by Synapse b.v. Plasmas spiked with thrombin calibrator were run in parallel with each cycle of test samples. The following parameters were analyzed using the appropriate software provided by Synapse b.v.: a) the lag time of thrombin generation, b) the time to reach the maximum concentration (Tmax) of thrombin, c) the maximum concentration (Cmax) of thrombin and d) the total amount of thrombin activity assessed as the area under the curve (i.e. the endogenous thrombin potential – ETP).

### Precision analysis of Thrombogram-Thrombinoscope assay

The intra-assay variability of each studied parameter of Thrombogram-Thrombinoscope was assessed by repeating the assay 6 times on the same assay plate for each studied dilution of recombiplastin and the inter-assay variability was assessed by repeating the assay 3 times, one after the other on different assay plates. Inter-individual variation was assessed by comparing the results obtained in 22 healthy subjects.

### Statistical analysis

Paired and unpaired Student's t-test were used to compare the mean values of TG parameters in frozen and not frozen PRP. SPSS statistical software package was used for statistical analysis. Values are depicted as means ± sd.

## Results

### Intra-assay variability of thrombogram

When TF was added in PRP, the thrombogram parameters showed acceptable intra-assay variability being equal or lower than 9%. The performance of the assay was independent of TF concentration (Table 1).

**Table 1:** Precision analysis of each parameter of thrombin generation curve given by Thrombogram-Thrombinoscope assessed in fresh PRP in the presence of increasing TF concentrations or with out any TF addition. CV: coefficient of variation.

Recombiplastin final dilution in plasma and approximate TF concentration	**Lag-time**		Tmax		**Cmax**		**ETP**	
	**intra-assay CV**	**inter-assay CV**	**intra-assay CV**	**inter-assay CV**	**intra-assay CV**	**inter-assay CV**	**intra-assay CV**	**inter-assay CV**

0 (0 pM)	7%	14%	5%	12%	5%	7%	4%	6%
1/2000 (3 pM)	4%	4%	4%	6%	6%	9%	4%	11%
1/1000 (6 pM)	4%	4%	3%	5%	6%	8%	3%	10%
1/500 (12 pM)	5%	6%	4%	6%	6%	8%	4%	5%
1/200 (30 pM)	6%	9%	5%	9%	3%	5%	4%	8%

### Inter-assay variability of thrombogram

All thrombogram parameters showed low inter-assay variability when thrombin generation was assessed in the presence of TF in PRP. Decreasing TF concentration did not significantly influence the inter-assay variability of the thrombogram parameters. In contrast, when no TF was added in the experimental system the inter-assay variability of the lag-time and the Tmax was considerably high (14% and 12% respectively). In contrast the inter-assay variability of the Cmax and the ETP was not significantly influenced by the absence of TF (Table 1).

The intra-assay and inter-assay coefficients of variation of the studied parameters were lower than 5% when thrombin generation was studied in PRP spiked with UFH (0.1 IU/ml to 1 IU/ml), or enoxaparin (0.1 anti-Xa IU/ml to 1 anti-Xa IU/ml) or fondaparinux (0,1 μg/ml to 1 μg/ml) in the presence of low TF concentration (final dilution of recombiplastin 1/2000).

### Inter-individual variability of thrombogram

Thrombogram parameters showed an important inter-individual variability and the coefficients of variation ranged from 18% to 45% being even more important when no TF was added. Among the studied parameters, the ETP and the Cmax showed the highest inter-individual variability mainly when no TF was added (Table 2).

**Table 2:** Inter-individual variability of thrombogram parameters in fresh PRP. Influence of TF concentration (n = 17). CV: coefficient of variations.

Recombiplastin final dilution in plasma and approximate TF concentration	Lag-time (% CV)	ETP (% CV)	Cmax (% CV)	Tmax (% CV)
0 (0 pM)	24%	31%	45%	25%
1/2000 (3 pM)	24%	25%	35%	24%
1/1000 (6 pM)	24%	25%	31%	24%
1/500 (12 pM)	22%	25%	27%	20%
1/200 (30 pM)	28%	25%	15%	17%

### Influence of TF concentration on thrombogram in fresh PPP and PRP

**Figure 1:**
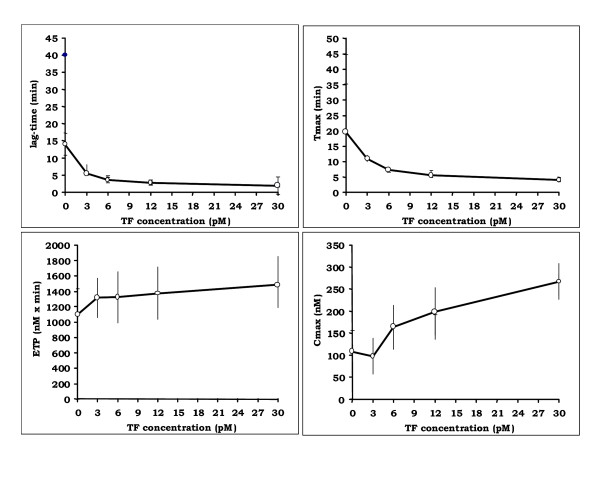
Effect of TF concentration of thrombin generation parameters in PRP from 17 individuals (values are means ± sd). According to the dosage of TF in RecomBiplasTin reagent performed in our laboratory, thromboplastin 1/1000 final dilution in plasma correspond to 6 pM final TF concentration.

Thrombin generation was triggered in fresh PRP from healthy individuals without any TF addition as well as in the presence of increasing TF concentrations (Figure 1). When thrombin generation was studied in PRP without any addition of TF a significant amount of thrombin was generated (Cmax = 108 ± 9 nM, ETP = 1094 ± 40 nM × min) with a lag-time of 14 ± 3.3 min and a Tmax of 19.5 ± 4.8 min. At recombiplastin dilution 1/2000, which corresponds to an approximate final concentration of TF of 3 pM, a threshold effect on the duration of the lag-time and the Tmax was observed. Increasing the concentration of TF resulted in an increase of the Cmax of thrombin. In contrast, the ETP was not influenced by the presence of TF, since the difference between the ETP values in the absence of TF and in the presence of recombiplastin dilution 1/2000 (which corresponds to approximately 3 pM final concentration of TF) was not statistically significant (1094 ± 340 nMxmin versus 1316 ± 255 nMxmin respectively; p > 0,05) (Figure 1). Table 3 depicts the normal values of Thrombogram parameters when coagulation is triggered in the presence of 1/1000 final dilution of recombiplastin (which corresponds to approximately 6 pM final concentration of TF).

**Table 3:** Normal values and inter-individual variability of thrombogram parameters in the presence of 1/1000 dilution of TF concentration in fresh PRP and in frozen-thawed PRP, (n = 22; mean ± sd).

Parameters of thrombin generation	**Fresh PRP**		Frozen-thawed PRP	
	
	Normal value	Inter-individual cv	Normal value	Inter-individual cv
Lag-time (min)	3.6 ± 0.8	24%	2	20%
ETP (nM × min)	1321 ± 330	25%	1550 ± 33	30%
Cmax (nM)	164 ± 50	31%	357 ± 1,66	25%
Tmax (min)	7.4 ± 1.8	24%	4,4 ± 0,1	25%

### Effect of platelet concentration on Thrombogram

The effect of platelet count on thrombin generation was studied in the presence of the lowest studied concentration of TF (1/2000 dilution of recombiplastin in plasma), which showed an acceptable precision of the assay.

The lag-time of thrombin generation was similar (8 ± 3 min) in the presence of platelet counts 10 × 10^9^/l and 50 × 10^9^/l. In the presence of platelet count 100 × 10^9^/l the lag-time was significantly shorter (5,8 ± 0,7 min) as compared to that observed in the presence of 50 × 10^9^/l (8 ± 3 min; p < 0,05). No difference of the lag-time was observed in platelet counts ranging from 100 × 10^9^/l to 400 × 10^9^/l (5.5 ± 0.5 min) (Table 4).

**Table 4:** Influence of platelet concentration on the normal values of thrombin generation parameters assessed by Thrombogram-Thrombinoscope in the presence of 1/2000 final dilution of recombiplastin corresponding to estimated TF concentration of 3 pM. (n = 7; means ± sd, *p < 0,05 versus the respective value in the presence of platelet counts 10 × 10^9^/l.

Platelet concentration (× 10^9^/l)	lag-time (min)	Tmax (min)	Cmax (nM)	ETP (nM × min)
400	5 ± 0,5	11 ± 2,7	161 ± 38	1633 ± 81
150	5,5 ± 0,5	11 ± 0,2	98 ± 40	1316 ± 255*
100	5,8 ± 0,7*	13 ± 0,9	72 ± 38	1135 ± 300
50	8 ± 3,3	12 ± 0,2*	63 ± 14*	1095 ± 301
10	8 ± 3	19 ± 2	43 ± 16	894 ± 100

The Tmax in the presence of platelet count 10 × 10^9^/l was 19 ± 2 min being significantly longer as compared to that observed in the presence of platelet count 50 × 10^9^/l (12 ± 0.2 min; p < 0.05). In the presence of platelet counts ranging from 50 × 10^9^/l to 400 × 10^9^/l the Tmax of thrombin generation did not significantly change (Table 4).

The Cmax of thrombin was significantly decreased in the presence of platelet count 10 × 10^9^/l, as compared to that observed in the presence of platelet count 50 × 10^9^/l (43 ± 16 nM and 63 ± 14 nM respectively; p < 0.05). In the presence of platelet concentrations ranging from 50 × 10^9^/l to 400 × 10^9^/l a slight but not statistically significant increase of Cmax was observed (Table 4).

In the presence of platelet count 10 × 10^9^/l the ETP was significantly lower as compared to that observed in the presence of platelet count 50 × 10^9^/l (894 ± 100 and 1095 ± 301 respectively; p < 0,05). In the presence of platelet counts ranging from 150 × 10^9^/l to 400 × 10^9^/l no significant differences of the ETP were observed (Table 4).

### Comparison of fresh and frozen-thawed platelet rich plasma for the assessment thrombogram

**Figure 2:**
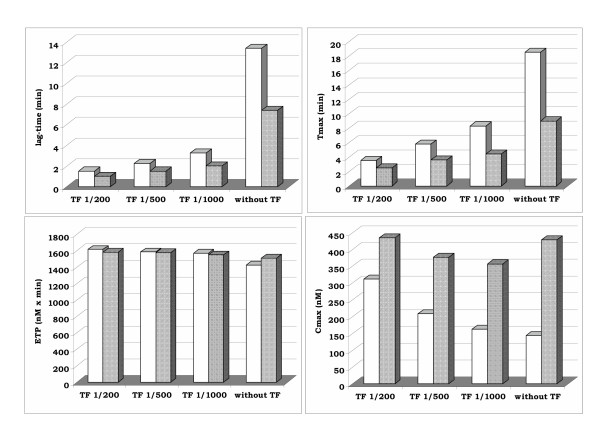
Thrombogram parameters in the presence of thromboplastin dilutions in fresh PRP (white bars) and frozen-thawed PRP (shadowed bars) (values are means of 4 experiments).

In thrombogram assessed in frozen-thawed PRP and in fresh PRP without any exogenous addition of TF both the lag-time and the Tmax were shorter by 45% and 51% and the Cmax of thrombin increased by 200% in frozen PPR as compared to the non frozen one (p < 0.05). The ETP was less affected by the freezing, since in frozen-thawed PRP it increased by only 6% as compared to that measured in fresh PRP (p > 0.05) (Figure 2).

In thrombogram assessed in the presence of TF, the ETP was the sole parameter, which was not significantly affected by the freezing (Figure 2). In frozen-thawed PRP, in the presence of each studied concentration of TF the lag-time decreased by 33% to 38% as compared to the non frozen one (p < 0.05). The Tmax was more markedly influenced by the freezing procedure mainly when minimal amounts of TF were employed. It was reduced by 47% in 1/1000 final dilution of recombiplastin in plasma, 38% in 1/500 final dilution of recombiplastin and 30% in 1/200 final dilution of recombiplastin as compared to the respective values in fresh PRP (p < 0.05). The freezing also affected the Cmax of thrombin, being significantly higher in frozen-thawed than in fresh PRP. The increase of the Cmax in frozen PRP was 119% in 1/1000 final dilution of recombiplastin, 81% in 1/500 final dilution of recombiplastin and39% in the presence of 1/200 final dilution of recombiplastin; in each case the difference was significant (p < 0.001) as compared to the respective values in fresh PRP.

The freezing procedure did not significantly influence the inter-individual variability of thrombogram assay when TF was added. In contrast freezing resulted in an important diminution of the intra-assay coefficient of variations of the ETP and Cmax when thrombogram was performed without addition of TF (Table 5).

**Table 5:** Intra-assay coefficients of variation of the studied parameters of thrombogram in fresh and frozen-thawed PRP (n = 4).

	**Lag-time**		**ETP**		**Cmax**		Tmax	
TF dilution	Fresh PRP	Frozen-thawed PRP	Fresh PRP	Frozen-thawed PRP	Fresh PRP	Frozen-thawed PRP	Fresh PRP	Frozen-thawed PRP

TF 1/200	0 %	0%	3,5%	2,6%	1%	0,7%	3,5%	0%
TF 1/500	0%	0%	3%	1,6%	1,6%	1,1%	4,1%	4%
TF 1/1000	0%	0%	2,1%	2,1%	5,2%	0,5%	2,9%	2,8%
without TF	3,6%	8,1%	4,2%	2,7%	9,8%	1,7%	4,3%	5,1%

The normal values and the inter-individual coefficient of variations of the studied parameters of thrombin generation after addition of 1/1000 final dilution of recombiplastin (approximately 6 pM TF) in fresh and in frozen-thawed PRP are summarized in Table 3.

### The influence of synthetic phospholipid concentration on thrombin generation in PPP

**Figure 3:**
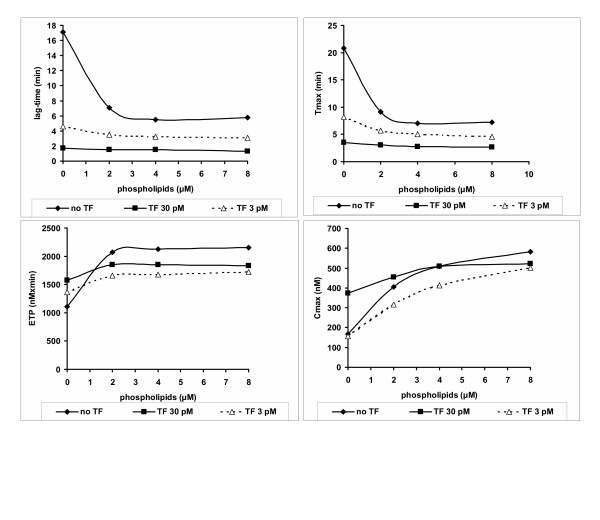
Influence of increasing concentrations of synthetic procoagulant phospholipids added in normal PPP. Thrombin generation was triggered in the presence of thromboplastin diluted in plasma 1/200 (30 pM TF) or 1/2000 (3 pM TF) or without any addition of TF. Values are means of 3 experiments.

The addition of synthetic procoagulant phospholipids in PPP strongly influenced all the parameters of thrombogram (Figure 3). Both the lag-time and the Tmax of thrombin generation were strongly dependent on the presence of procoagulant phospholipids when no TF was added. In the presence of 3 pM of TF both parameters were influenced to a less significant degree by the concentration of phospholipids. In contrast the impact of the procoagulant phospholipids was important on both the ETP and the Cmax of thrombin either in the absence of TF or in the presence of low (3 pM) or higher concentrations (30 pM). For all the parameters of thrombogram a threshold effect was observed in the presence of phsopholipid concentrations equal or higher to 4 μM. However, since the Cmax appeared a slight, though not significant increase in the presence of synthetic phospholipid concentration of 8 μM, the optimum concentration of phospholipids is situated at 8 μM.

## Discussion

In the present study thrombin generation was assessed with the automated Thrombogram-Thrombinoscope assay. The precision of thrombogram, was evaluated in the presence of increasing TF concentrations, of synthetic procoagulant phospholipids, platelet count and freezing – thawing of platelet rich plasma from healthy volunteers. Normal values of thrombin generation in defined experimental, conditions which can be applied in routine haemostasis laboratory have also been established.

In the presence of a standardized platelet concentration (150 × 10^9^/l) without any addition of TF all the studied parameters of thrombin generation can be measured. Thus we confirm that the presence of procoagulant phospholipids derived from platelets as in our study, is of major importance for steady thrombin generation [22]. The presence of platelets as is the case in our study, or the in vitro addition of negatively charged phospholipids in PPP [20] creates a more physiologically relevant experimental system.

The amplification of thrombin generation also depends on the concentration of the initial stimulus. According to the old scheme of blood coagulation (the cascade scheme) [23,24] one can trigger either the TF pathway or the contact system (intrinsic pathway) in order to study thrombin generation. However, the classic cascade scheme has a limited physiological relevance. It is now generally accepted that tiny amounts of TF, either blood born (from microparticles or activated monocytes) or exposed at the site of vascular injury, bind to FVII and FVIIa and the complex TF/FVIIa triggers blood coagulation [25]. Therefore we validated the Thrombogram-Thrombinoscope assay in TF-induced coagulation of normal plasma. As it was expected, in PRP the lag-time of thrombin generation onset, the Cmax of thrombin and the Tmax were strongly influenced by the TF concentration. The values of the lag-time, Cmax, Tmax and ETP in PRP did not significantly vary when physiologically relevant concentrations of TF, ranging from 1/2000 to 1/1000 dilution of recombiplastin, were used (which correspond to approximately 3 pM to 6 pM of TF).

Thrombogram assay performed in the presence of TF concentrations (3 pM to6 pM) which are considered to be physiologically relevant, had a low inter-assay and intra-assay coefficients of variation in PRP revealing that the precision of the assay is marginally influenced by the concentration of TF. However, all the studied parameters of thrombogram performed in PRP showed an important inter-individual variability, which was not improved by the addition of TF. Chantarangkul et al [20] showed that the presence of residual platelets into the PPP influences the imprecision of the ETP measurement. Our study shows that the inter-individual variability is important in fresh PRP, as well as in frozen-thawed PRP and is not influenced when the platelet count ranges from 10 × 10^9^/l to 400 × 10^9^/l (data not shown). A significant individual dependent variation in thrombogram has also been reported by Vanschoonbeek et al [26]. Thus it seems that individual functional characteristics of platelets, which are not completely understood today, as well as variations of the concentration of clotting factors and the natural inhibitors of blood coagulation [27] influence thrombin generation profile and they can be detected by the thrombogram assay. Platelet debris or microparticles present in PPP might have a major contribution to the observed significant interindividual variability of thrombin generation assay either in the absence or in the presence of TF since they exert a "non standardized" procoagulant activity. In order to eliminate this unpredictable factor it has been proposed that complete platelet depletion (i.e. by filtration of PPP) or addition of an excess concentration of phospholipids in PPP may improve the inter-individual variability of thrombogram [20]. We have to stress out that in the presence of very low TF concentration (3 pM) or in its absence, the CV values of the interindividual variability of thrombin's Cmax are considerably high. This finding allows to postulate that in conditions where the initial trigger of coagulation process is weak the experimental system is quite unstable. In such conditions the impact of artifacts such as platelet debris or probably the presence of procoagulant material such as microparticles is more important. It has been widely recognized that the major contribution of the Thrombogram-Thrombinoscope methodology is that it allows the study of thrombin generation in a more physiologically relevant system where platelets and fibrinogen/fibrin are present. Whether it is better to omit platelets from the experimental system has to be controlled in more detailed studies in larger groups of normal individuals and of patients with hyper- or hypo-coagulant states. In any case the large inter-individual variation could make more difficult the interpretation of the results.

Another important aspect of Thrombogram-Thrombinoscope assay is that the studied parameters are not significantly influenced by quantitative platelet variations within a quite wide range of platelet concentration (from 50 × 10^9^/l to 400 × 10^9^/l). As we have described in details elsewhere [28] and in accordance with the findings from Chantarangkul et al [20] thrombin generation is significantly reduced when platelet count is lower than 50 × 10^9^/l. Consequently, deviations from the normal platelet concentrations do not significantly influence neither the precision of the assay nor the reference normal values.

Freezing procedure of PRP significantly reduced the lag-time and the Tmax and also increased the Cmax of generated thrombin but it did not significantly affect the ETP. Thus in frozen-thawed PRP thrombin generation is accelerated and the maximum amount of generated thrombin is increased apparently due to cold-induced platelet activation and/or procoagulant phospholipids release. However, the integral amount of thrombin generated in time, expressed by the ETP is not modified. In addition, freezing does not modify the coefficients of variation of all the studied parameters of thrombogram when thrombin generation was triggered in the presence of TF showing once more that the high interindividual variability of the assay is poorly related to technical conditions. Consequently ETP is the single parameter of Thrombogram which can be assessed in frozen-thawed PRP but weather its sensitivity to detect pathological hypercoagulable or hypocoagulable conditions needs to be examined. From a practical point of view, when clotting is triggered in PPP spiked with synthetic procoagulant phospholipids at concentrations higher than 4 μM in the presence of low TF concentration (1/2000 recombiplastin dilution in plasma) results in a similar pattern of thrombogram as that observed when PRP was used. In addition the ensemble of the presented data show that the ETP is influenced more by the concentration of procoagulant phospholipids rather than by the concentration of TF.

In conclusion, the present study shows that Thrombogram-Thrombinoscope assay performed in fresh platelet rich plasma has an acceptable precision, with low inter-assay and intra-assay coefficient of variations. The concentration of TF is determinant for the normal values of all the studied parameters except the endogenous thrombin potential. The addition of physiologically relevant concentrations of TF in platelet rich plasma (1/1000 to 1/2000 final dilution of recombiplastin) simulates a more natural experimental system from which however, the white and red blood cell as well as the rheological conditions are absent. Assessing thrombin generation in frozen-thawed PRP induces an important bias on the normal values of all the parameters of thrombogram except the ETP. In contrast, the thrombogram parameters obtained in frozen-thawed PPP supplemented with synthetic procoagulant phospholipids at concentrations equal or superior than 4 μM and physiologically relevant concentrations of TF is quite similar to that obtained in PRP in the presence of the same TF concentration. Regarding all the parameters of thrombin generation, the optimal concentration of the synthetic phospholipids employed in the present study is situated at 8 μM. The use of a standardized thrombin calibrator, which runs in parallel with the studied samples, allows the quantitative expression of thrombin in nM and ETP in nM × min. In addition, thrombogram software can reliably describe the distinct phases of thrombin generation (i.e the initiation phase, the Tmax and the maximum concentration of the generated thrombin). Among the studied parameters of thrombin generation given by the Thrombogram-Thrombinoscope assay, the ETP is less influenced by the concentration of TF as well as by the pre-analytical conditions (fresh PRP or frozen-thawed PRP) but it is strongly influenced by the concentration of phospholipids when it is performed in PPP. The influence of commercially available citrate preparations on thrombogram has to be evaluated in future studies. Thrombogram-Thrombinoscope assay is sensitive to the presence of UFH, enoxaparin and fondaparinux with low intra-assay and inter-assay variability. However, we have shown that the lag-time and the velocity of the propagation phase are more relevant than the ETP when an indirect FXa inhibitor (fondaparinux) or rFVIIa are present in therapeutic concentrations [3,28]. The method can be useful for the study of different antithrombotic agents whatever their mechanism of action and their target (LMWHs, direct and indirect inhibitors of FXa and direct inhibitors of thrombin [3,17,29,30]. Thus the ensemble of the parameters of thrombin generation can be potentially used for the laboratory diagnosis and biological of a large spectrum of hyper- and hypo-coagulant states. The high inter-individual variability of thrombogram is a topic that has to be studied in details in a large collaborative study.

## List of abbreviations

Cmax: maximum concentration of thrombin

CV: coefficient of variation

ETP: endogenous thrombin potential

LMWH: Low Molecular Weight Heparin

PPP: Platelet Poor Plasma

PRP: Platelet Rich Plasma

TF: Tissue Factor

Tmax: time required to reach the maximum concentration of generated thrombin

## Authors' contributions

**GTG **conceived of the study, organized its design and coordination, interpreted the data and drafted the manuscript. **FD **contributed to the design of the study and interpretation of the data. **JB **participated in the design of the study, the acquisition of the data and the statistical analysis. **LL **participated in the experiments with the synthetic phospholipids. **IE **participated to the critical reading of the manuscript. **MMS **participated to the design and coordination of the study, the interpretation of the data and contributed to the drafting of the manuscript.
